# Association of systemic immune inflammation index and system inflammation response index with clinical risk of acute myocardial infarction

**DOI:** 10.3389/fcvm.2023.1248655

**Published:** 2023-08-30

**Authors:** Xing Wei, Zhipeng Zhang, Jing Wei, Chunmiao Luo

**Affiliations:** ^1^ Department of Cardiology, The Second People's Hospital of Hefei, Hefei Hospital Affiliated to Anhui Medical University, Hefei, China; ^2^ The Fifth Clinical School of Medicine, Anhui Medical University, Hefei, China

**Keywords:** acute myocardial infarction, systemic immune inflammation index, system inflammation response index, MACE, Gensini, QTc, GRACE

## Abstract

**Background:**

Systemic immune inflammatory index (SII) and systemic inflammatory response index (SIRI) are combinations of non-specific inflammatory and adaptive immune response impairments associated with cardiovascular disease. Yet little analysis has been done on SII, SIRI and acute myocardial infarction (AMI) prognosis. The purpose of this study was to investigate the correlation of SII and SIRI with clinical risk factors such as GRACE, Gensini, and QTc after acute myocardial infarction.

**Methods:**

This study enrolled 310 patients with AMI from February 1, 2018, to December 31, 2022, at our institution. Routine blood items calculated SII and SIRI. Two groups were divided according to whether MACE occurred: the MACE group (81 cases) and the NMACE group (229 cases); each group was divided into three groups according to the SII and SIRI tertiles. The relationship between SII, SIRI and MACE was analyzed using multifactorial logistic regression analysis after adjusting for confounders; ROC curves were plotted to examine the predictive value of SII and SIRI for MACE. The correlation between SII and SIRI and potential risk factors such as Gensini, QTc and GRACE was further analyzed.

**Results:**

The study enrolled 310 patients, comprising 248 men (80%, mean age 60.73 ± 13.695 years) and 62 women (20%, mean age 69.79 ± 11.555 years). In the regression model completely adjusted for confounders, the risk of MACE was higher in AMI patients with SII > 11.00 [OR = 1.061,95% CI (1.018,1.105)] than in SII < 5.98; the risk of MACE was 115.3% higher in AMI patients with SIRI (1.72–3.68) [OR = 2.153, 95% CI (1.251, 3.705)] was 115.3% higher in AMI patients with SIRI < 1.72 and the risk of MACE was 25.1% higher in AMI patients with SIRI > 3.68 [OR = 1.251, 95% CI (1.123, 1.394)] than in AMI patients with SIRI < 1.72. In addition, SII, SIRI, and potential post-infarction risk factors (Gensini, QTc, and GRACE) were also associated.

**Conclusion:**

SII and SIRI have been significantly associated with post-myocardial infarction MACE and the predictive potential clinically integrated risk factors in AMI patients, for which more attention should be paid to targeted anti-inflammatory therapy in AMI patients to further reduce the incidence of prognostic MACE in AMI patients.

## 1. Introduction

Over the years, cardiovascular disease (CVD) outcomes have improved substantially, but CVD is still the leading cause of mortality in the global community (1). The occurrence and development of CVD are mainly attributed to atherosclerosis and thrombosis-induced narrowing of blood vessels, which ultimately leads to end-organ dysfunction characterized by myocardial infarction (2).

Acute myocardial infarction (AMI) is the ultimate phase of atherosclerotic cardiovascular disease, for which prognosis management is the critical element of patients' quality of life (3). Numerous pieces of research have demonstrated that AMI patients are frequently followed by major adverse cardiovascular events (MACE) such as acute heart failure (4), malignant arrhythmias (5–7), cardiogenic shock (8, 9) and sudden cardiac death (10) after PCI. Briefly, assessing risk after PCI in patients with AMI is particularly important.

The Global Registry of Acute Coronary Events (GRACE) score is an assessment system of risk based on risk factors for patients with acute coronary syndromes to evaluate mortality risk in patients with ACS within six months of hospital admission (11). Previous studies have indicated that the GRACE score could be utilized to assess the early risk of non-ST-segment elevation myocardial infarction and acute myocardial infarction (12). The Gensini score is a valid tool for evaluating the severity of coronary artery disease (CAD) by coronary imaging features with high predictive value for long-term outcomes in patients with coronary artery disease undergoing PCI (13). The QTc interval visually reflects changes in ventricular repolarization, of which prolonged QTc interval is considered an essential element associated with ventricular arrhythmias (14). The GRACE score, Gensini score, and QTc combine to reflect the potential risk of patients with AMI.

The Systemic Immune Inflammation Index (SII) and the Systems Inflammation Response Index (SIRI) involve numerous well-known markers of inflammation, reflecting the balance between inflammation and immune response (15). Research has demonstrated that it accurately predicts poor prognosis in patients with gastrointestinal malignancies (16, 17). SII and SIRI are associated with the incidence of coronary artery lesions and acute coronary syndromes in patients with coronary atherosclerotic heart disease (15). Nevertheless, few studies have been conducted on the correlation of SII and SIRI with the prognosis of AMI patients and potential risk factors such as GRACE score, Gensini score, and QTc.

The present study aimed to evaluate SII and SIRI in AMI patients who developed post-infarction MACE and further explored the correlation between SII and SIRI and clinical risk in AMI patients.

## 2. Materials and methods

### 2.1. Baseline information on the study population

A total of 310 AMI patients who underwent coronary angiography and interventional therapy at our hospital from February 1, 2018 to December 31, 2022 were included in this study, which included 248 males (average age 60.73 ± 13.69 years) and 62 females (average age 69.79 ± 11.55 years). According to the occurrence of mace, the patients were divided into the MACE group (81 cases, 26.13%) and the NMACE group (229 cases, 73.87%). The patients were divided into three groups using the SII and SIRI triad: 1. Q1 (*n* = 103, SII < 598.01), Q2 (*n* = 104, 598.01 ≤ SII < 1,100.12), Q3 (*n* = 103, 1,100.12 ≤ SII); 2. Q1 (*n* = 103, SIRI < 1.72), Q2 (*n* = 104, 1.72 ≤ SIRI < 3.68), Q3 (*n* = 103, 3.68 ≤ SIRI). The general demographics of the sample included age, sex, history of smoking, previous hypertension, and history of diabetes mellitus. Enrollment criteria for this investigation were a precise diagnosis of AMI according to the appropriate guidelines (based on myocardial necrosis markers such as cTnI, CK-MB or high-sensitivity troponin and ECG presentation) (18). Exclusion criteria: the recent history of major surgery, severe renal failure or liver function abnormalities, contrast allergy, history of malignancy, aortic coarctation, and lack of documented clinical data.

MACE was defined as sudden cardiac death, cardiogenic shock, acute left heart failure, cerebral infarction, cerebral haemorrhage, and in-hospital first malignant arrhythmia (ventricular tachycardia, ventricular fibrillation, ventricular arrest, and third-degree AV block). SII was defined as (neutrophil count) × (platelet count)/(lymphocyte count). SIRI was calculated as (neutrophil count) × (monocyte count)/(lymphocyte count) (15, 16).

### 2.2. Laboratory metrics and imaging

Venous blood samples were obtained from the elbow vein of each patient in the early morning on an empty stomach. Routine haematological indicators were measured, which included neutrophils, lymphocytes, monocytes, haemoglobin (HGB), platelets (PLT), and serum biochemical indicators such as triacylglycerol (TG), total cholesterol (TC), high-density lipoprotein cholesterol (HDL-C), low-density lipoprotein cholesterol (LDL-C), creatinine, fasting glucose (Glu), homocysteine (Hcy). ECG indexes such as heart rate, QT/QTc and cardiac ultrasound indexes of left ventricular ejection fraction (LVEF) were collected at the time of patient admission.

### 2.3. Coronary intervention

The patients with AMI were administered 300 mg of aspirin + 180 mg of ticagrelor or 300 mg of aspirin + 225 mg of clopidogrel as a loading dose antiplatelet agent directly on admission. Selective coronary angiography was obtained using the standard Judkin technique in all study group members, with left coronary angiography results read in the selected head, foot, right shoulder and spider views, and right coronary angiography results read in described left and head views. Coronary stenosis was evaluated according to the Genisi score. Two interventional cardiologists read all coronary angiograms.

### 2.4. Statistical analysis

The present study used SPSS 26.0 and R 4.2.3 for statistical analysis and GraphPad Prism 9.0 for graphical plotting. Normality was tested for continuous numerical variables using the Shapiro-Wilk test, and non-normally distributed data were expressed as median (P25, P75). The patients were separated into the MACE group (81 cases) and the NMACE group (229 cases) according to whether they had MACE after PCI. Non-normally distributed data were indicated as median (P25, P75), and the Mann-Whitney *U*-test was conducted for comparison between groups; data conforming to a normal distribution were stated as mean ± standard deviation and compared between the two groups using the independent samples *t*-test; categorical variables were represented as percentages, and the chi-square test was utilized for comparison between groups. In order to eliminate the effect of confounding factors, this study conducted a propensity score matching method to study the differences in SII and SIRI between the two groups.

Further tertiles were performed from lowest to highest according to SII and SIRI, and group comparisons were performed between the three groups using one-way ANOVA, chi-square test and nonparametric rank sum test. The Q1 group was treated as the reference, followed by univariate and multivariate logistic regression analysis with Q2 and Q3 groups, respectively. The least adjusted model was modified by sex, age, smoking history, diabetes, hypertension, and Killip classification, and the fully regulated model was modified by adding LVEF, monocytes, platelets, creatinine, HDL-C, LDL-C, triglycerides, cholesterol, fasting glucose, and Hcy based on the least adjusted model. The median of the three groups of SII and SIRI were taken for covariate setting and tested for trend. Subject work (ROC) curves were plotted to analyze the predictive value of SII and SIRI for MACE, and the sensitivity and specificity of the predictors were determined using the maximum of Youden's index. The Delong nonparametric test was used to analyze whether there was a difference in the predictive value of individual and the joint indicator for MACE. Spearman correlation analysis was used to assess whether there was a linear correlation between SII and SIRI and GRACE score, Gensini score, and QTc. Statistically significant with *P* value < 0.05.

## 3. Results

### 3.1. Comparison of clinical indicators between the MACE and NMACE groups

Three hundred and ten samples were included in this study, comprising 248 men (80%, average age 60.73 ± 13.695 years) and 62 women (20%, average age 69.79 ± 11.555 years). As shown in Table 1, the MACE group was older and had a higher Killip classification compared to the NMACE group (*P *< 0.001). LVEF and HGB were lower in the MACE group than in the NMACE group (*P *< 0.05); neutrophils, monocytes, lymphocytes, creatinine, fasting glucose, Hcy, SII, SIRI, Gensini, and GRACE were higher than those in the NMACE group (*P *< 0.05). In the present study, propensity scores were matched on the basis of confounding factors such as age, LVEF, QTc, HGB, creatinine, fasting glucose, Hcy and Killip classification. As shown in Supplementary Table S1, the difference in SII between the two groups was statistically significant (*P *< 0.05), while the difference in SIRI between the two groups was not statistically significant (*P *> 0.05).

**Table 1:** Comparison of baseline characteristics of the study subjects based on MACE.

Features	MACE (*n* = 81)	NMACE (*n* = 229)	X^2^/Z/T value	*P*
Male, *n* (%)	63 (77.78)	185 (80.79)	0.338	0.561
Age (years)	67.06 ± 13.60	60.94 ± 13.48	−3.501	0.001
Diabetes, *n* (%)	30 (37.04)	65 (28.38)	2.108	0.147
Hypertension, *n* (%)	52 (64.20)	127 (55.46)	1.873	0.171
Smoking, *n* (%)	40 (49.38)	125 (54.59)	0.650	0.420
Killip(Ⅱ-Ⅳ), *n* (%)	50 (61.73)	59 (25.76)	33.947	<0.001
LVEF (%)	55.00 (48.00, 60.00)	60.00 (56.00, 65.00)	−4.726	<0.001
QTc (ms)	461.00 (430.00, 493.00)	432.00 (414.00, 452.00)	−5.936	<0.001
Neutrophils (×10^9^/L)	8.42 (6.24, 11.85)	6.22 (4.60, 8.16)	−5.813	<0.001
Monocyte (×10^9^/L)	0.70 (0.40, 0.93)	0.60 (0.40, 0.80)	−2.195	0.028
Lymphocytes (×10^9^/L)	1.30 (0.99, 1.96)	1.56 (1.17, 2.07)	−2.200	0.028
HGB (g/L)	128.00 (115.50, 142.50)	135.00 (123.00, 146.50)	−2.527	0.011
PLT (×10^9^/L)	192.00 (137.00, 231.50)	189.00 (153.50, 225.40)	−0.470	0.638
Creatinine (μmol/L)	80.10 (68.00, 100.35)	70.40 (60.00, 84.00)	−3.206	0.001
TG (mmol/L)	1.39 (0.98, 1.98)	1.52 (1.08, 2.39)	−1.514	0.130
TC (mmol/L)	4.20 (3.57, 5.13)	4.29 (3.67, 4.97)	−0.115	0.908
HDL-C (mmol/L)	1.09 (0.96, 1.32)	1.05 (0.90, 1.20)	−1.504	0.133
LDL-C (mmol/L)	2.73 (2.08, 3.45)	2.73 (2.16, 3.31)	−0.014	0.989
Glu (mmol/L)	6.88 (5.81, 9.02)	5.97 (5.14, 7.61)	−3.474	0.001
Hcy (μmol/L)	17.80 (13.25, 20.97)	13.70 (11.00, 17.60)	−3.846	<0.001
SII	1,255.89 (681.18, 2,190.82)	747.95 (455.30, 1,150.52)	−4.770	<0.001
SIRI	4.07 (2.42, 7.65)	2.13 (1.28, 3.81)	−5.528	<0.001
Gensini	81.00 (50.00, 101.50)	47.00 (35.00, 64.00)	−6.805	<0.001
GRACE	135.50 (108.50, 157.45)	96.30(80.90, 120.15)	−7.702	<0.001

Normally distributed continuous variables are described by mean ± standard deviation, non-normally distributed continuous variables are expressed as median (interquartile range), and categorical variables are expressed as numbers (percentages).

Killip class, clinical classification of heart failure; LVEF, left ventricular ejection fraction; HGB, haemoglobin; PLT, platelets; TG, triglycerides; TC, total cholesterol; HDL-C, high-density lipoprotein cholesterol; LDL-C, low-density lipoprotein cholesterol; Glu, fasting glucose; Hcy, homocysteine; SII, Systemic Immune Inflammation Index; SIRI, System Inflammation Response Index; Gensini, coronary stenosis score; GRACE, global registry of acute coronary events score.

### 3.2. ROC curve analysis of SII and SIRI for MACE

**Figure 1:**
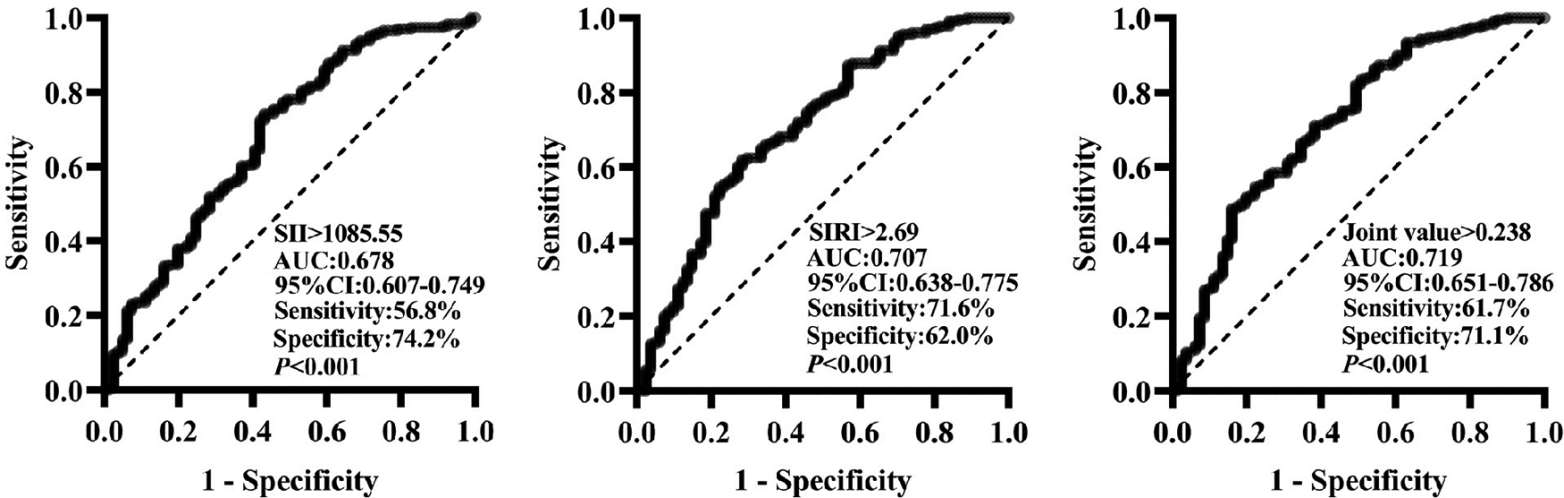
Predictive value of SII and SIRI for MACE (ROC curve analysis). SII, systemic inflammatory index; SIRI, system inflammation response index; AUC, area under the curve; 95% CI, 95% confidence interval.

The ROC curve was utilized for analysis in the present study to clarify the predictive value of SII and SIRI for MACE. As shown in Figure 1, the predictive value (AUC) of SII for MACE was 0.678 [95%CI (0.607–0.749)], with the highest predictive value at the cut-off value of 1,085.55, and its sensitivity was 56.8%, and specificity was 74.2% (*P *< 0.001); the predictive value (AUC) of SIRI for MACE was 0.707 [95%CI (0.638–0.775)], with the highest predictive value at a cut-off value of 2.69, its sensitivity of 71.6% and specificity of 62.0% (*P *< 0.001). The joint predictive value of SII and SIRI was highest when the cutoff value was >0.238 [AUC = 0.719, 95%CI (0.651–0.786), *P *< 0.001]. The delong nonparametric test analysis showed that the joint predictive value of SII and SIRI was higher than the SII,which was statistically different (*P* = 0.036); however, there was no statistically significant difference between the predictive value of SII and SIRI (*P* > 0.05).

### 3.3. Correlation analysis of SII and SIRI with comprehensive post-myocardial infarction clinical risk factors of patients (linear analysis)

**Figure 2:**
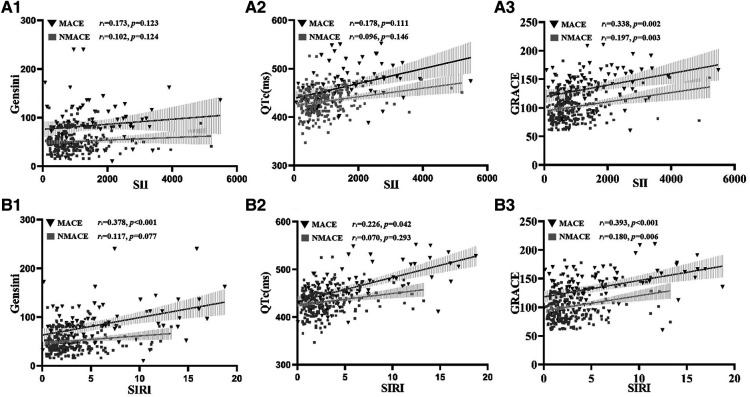
Correlation analysis of SII and SIRI with Gensini, QTc, and GRACE. SII, systemic inflammatory index, SIRI, System inflammation response index, Gensini, coronary stenosis score, GRACE, global registry of acute coronary events score, r, correlation coefficient. The MACE group showed a linear correlation between SII and GRACE (*r*=0.338, *P*=0.002) (**A**_3_), SIR was linearly correlated with Gensin (*r*=0.378, *P*<0.001) (**B**_1_), QTc (*r*=0.226, *P*=0.042) (**B**_2_), and GRACE (*r*=0.393, *P*<0.001) (**B**_3_). the NMACE group showed a linear correlation between SII and GRACE were linearly correlated (*r*=0.197, *P*=0.003) (**A**_3_) and SIRI was linearly correlated with GRACE (*r*=0.180, *P*=0.006) (**B**_3_).

In the MACE group, SII was positively correlated with GRACE (*r* = 0.338, *P *= 0.002); SIRI was positively correlated with Gensini (*r* = 0.378, *P *< 0.001), QTc (*r* = 0.226, *P *= 0.042) and GRACE (*r* = 0.393, *P *< 0.001). In the NMACE group, SII was positively correlated with GRACE (*r* = 0.197, *P *= 0.003); SIRI was positively correlated with GRACE (*r* = 0.180, *P *= 0.006) (Figure 2).

### 3.4. Comparison of baseline information based on SII and SIRI triple quantile

The members of the study group were separated equally into three groups according to the tertiles of SII: group Q1 (<598.01), group Q2 [598.01–1,100.12], and group Q3 (>1,100.12). As shown in Table 2, high levels of the SII group had higher Killip grade and were associated with lower LVEF and TG and with higher QTc, monocytes, TC, HDL-C, LDL-C, and Glu. There appears to be a strong association between elevated levels of SII and the initiation of in-hospital MACE. Similarly, the patients were divided into three groups according to the SIRI triple quantile method: group Q1 (<1.72), group Q2 [1.72–3.68], and group Q3 (>3.68). As shown in Table 3, high levels of the SIRI group were associated with elevated QTc and Glu and high Killip grade and remained with reduced LVEF and TG levels. In the postoperative evaluation of the combined risk factors, both the Q2 and Q3 groups had higher GRACE and Gensini levels compared to the Q1 group in the intergroup comparison based on the SII and SIRI tertile. (Considering the calculation of SII and SIRI, the comparison of neutrophils, platelets, and lymphocytes was not included in the group comparison of SII in this investigation, while the comparison of neutrophils, monocytes, and lymphocytes was eliminated in the group comparison of SIRI).

**Table 2:** Comparison of baseline characteristics based on SII triple quantile.

Features	SII triple quantile			*P*
	Q1 (*n* = 103)	Q2 (*n* = 104)	Q3 (*n* = 103)	
Male, *n* (%)	83 (80.58)	85 (81.73)	80 (77.67)	0.753
Age (years)	61.02 ± 13.43	62.62 ± 12.50	63.98 ± 15.20	0.304
Diabetes, *n* (%)	28 (27.18)	36 (34.62)	31 (30.09)	0.505
Hypertension, *n* (%)	58 (56.31)	63 (60.57)	58 (56.31)	0.773
Smoking, *n* (%)	60 (58.25)	48 (46.15)	57 (55.34)	0.190
Killip (Ⅱ-Ⅳ) *n* (%)	31 (30.09)	26 (25.00)	52 (50.48)	<0.001
LVEF (%)	60.00 (55.00, 65.00)	61.50 (56.00, 66.00)	56.00 (48.00, 60.00)	<0.001
QTc (ms)	431.00 (415.00, 458.00)	435.00 (413.25, 451.50)	451.00 (424.00, 473.00)	<0.001
Monocyte (×10^9^/L)	0.57 (0.40, 0.70)	0.60 (0.41, 0.80)	0.70 (0.40, 1.00)	0.015
HGB (g/L)	133.00 (123.00, 143.00)	134.50 (119.25, 143.75)	132.00 (118.00, 149.00)	0.987
Creatinine (μmol/L)	74.59 ± 18.22	75.60 ± 22.70	76.02 ± 22.53	0.885
TG (mmol/L)	1.62 (1.17, 2.49)	1.41 (1.01, 1.95)	1.36 (0.91, 2.08)	0.010
TC (mmol/L)	4.11 (3.46, 4.68)	4.27 (3.78, 5.03)	4.54 (3.67, 5.28)	0.013
HDL-C (mmol/L)	1.02 (0.88, 1.15)	1.10 (0.91, 1.20)	1.09 (0.96, 1.31)	0.024
LDL-C (mmol/L)	2.62 (1.95, 3.22)	2.76 (2.16, 3.30)	2.82 (2.19, 3.55)	0.050
Glu (mmol/L)	5.67 (4.98, 7.22)	6.33 (5.16, 8.40)	6.52 (5.74, 8.89)	0.002
Hcy (μmol/L)	15.20 (12.50, 18.69)	14.15 (10.74, 18.00)	14.27 (10.80, 19.70)	0.361
Gensini	48.00 (35.00, 60.00)	48.00 (36.00, 81.20)	60.00 (38.50, 88.00)	0.001
GRACE	96.40 (81.20, 116.50)	107.25 (81.42, 123.70)	127.30 (89.60, 152.70)	<0.001
MACE *n* (%)	16(15.53)	21(20.19)	44(42.72)	<0.001

Normally distributed continuous variables are described by mean ± standard deviation, non-normally distributed continuous variables are quantified as median (interquartile range), and categorical variables are quantified as numbers (percentages).

Killip class, clinical classification of heart failure; LVEF, left ventricular ejection fraction; HGB, haemoglobin; TG, triglycerides; TC, total cholesterol; HDL-C, high-density lipoprotein cholesterol; LDL-C, low-density lipoprotein cholesterol; Glu, fasting glucose; Hcy, homocysteine; SII, systemic inflammatory index; Gensini, coronary artery stenosis score; GRACE, global registry of acute coronary events score.

**Table 3:** Comparison of baseline characteristics based on SIRI triple quantile.

Features	SIRI triple quantile			*P*
	Q1 (*n* = 103)	Q2 (*n* = 104)	Q3 (*n* = 103)	
Male, *n* (%)	78 (75.73)	83 (79.81)	87 (84.47)	0.292
Age (years)	60.66 ± 12.93	62.81 ± 13.97	64.16 ± 14.26	0.185
Diabetes, *n* (%)	32 (31.07)	31 (29.81)	32 (31.07)	0.975
Hypertension, *n* (%)	59 (57.28)	63 (60.58)	57 (55.34)	0.743
Smoking, *n* (%)	57 (55.34)	56 (53.85)	52 (50.49)	0.774
Killip (Ⅱ-Ⅳ) *n* (%)	33 (32.04)	28 (26.92)	48 (46.60)	0.009
LVEF (%)	60.00 (56.00, 65.00)	60.00 (55.00, 64.00)	56.00 (49.00, 62.00)	<0.001
QTc (ms)	435.00 (418.00, 454.00)	430.50 (409.75, 456.00)	449.00 (427.00, 472.00)	<0.001
PLT (×10^9^/L)	178.00 (145.00, 212.00)	195.00 (157.00, 227.75)	189.00 (151.00, 234.00)	0.220
HGB(g/L)	133.00 (123.00, 146.00)	132.00 (118.00, 143.00)	132.00 (118.00, 147.00)	0.573
Creatinine (μmol/L)	73.00 (64.00, 88.00)	71.00 (59.25, 86.60)	76.00 (59.00, 92.80)	0.367
TG (mmol/L)	1.54 (1.17, 2.45)	1.42 (0.97, 2.31)	1.39 (1.00, 1.98)	0.028
TC (mmol/L)	4.29 (3.53, 4.89)	4.13 (3.52, 5.02)	4.40 (3.72, 5.19)	0.193
HDL-C (mmol/L)	1.04 (0.90, 1.20)	1.06 (0.89,1.19)	1.10 (0.98, 1.31)	0.064
LDL-C (mmol/L)	2.75 (2.08, 3.31)	2.51 (2.11, 3.27)	2.78 (2.27, 3.43)	0.298
Glu (mmol/L)	5.69 (5.02, 7.61)	6.24 (5.12, 8.13)	6.61 (5.77, 8.89)	0.005
Hcy (μmol/L)	14.27 (11.81, 17.60)	15.19 (11.22, 18.27)	15.31 (10.61, 20.90)	0.938
Gensini	47.00 (34.00, 60.00)	48.00 (35.00, 72.00)	64.00 (42.00, 96.00)	<0.001
GRACE	96.40 (83.30, 116.50)	103.75 (80.95, 126.02)	127.30 (93.60, 151.70)	<0.001
MACE *n* (%)	14(13.59)	23(22.11)	44(42.72)	<0.001

Normally distributed continuous variables are described by mean ± standard deviation, non-normally distributed continuous variables are expressed as median (interquartile range), and categorical variables are expressed as numbers (percentages).

Killip class, clinical classification of heart failure; LVEF, left ventricular ejection fraction; PLT, platelets; HGB, haemoglobin; TG, triglycerides; TC, total cholesterol; HDL-C, high-density lipoprotein cholesterol; LDL-C, low-density lipoprotein cholesterol; Glu, fasting glucose; Hcy, homocysteine; SIRI, System Inflammation Response Index; Gensini, coronary stenosis score; GRACE, global registry of acute coronary events score.

### 3.5. Regression analysis and trend test for each group under different adjustment models based on SII and SIRI triple quartiles

The risk of MACE in the SIIQ3 group was increased by 7.9% [OR = 1.079,95%CI (1.043–1.115), *P *< 0.001] in the unadjusted model with the SIIQ1 group as control; the risk of MACE was increased by 50.2% in the SIRIQ2 and SIRIQ3 groups, compared with the SIRIQ1 group as control [OR = 1.502,95%CI (1.018–2.216), *P *< 0.05] and 28.3% [OR = 1.283,95%CI (1.173–1.404), *P* < 0.001]. In model 1, the risk of MACE was increased by 6.5% in the SIIQ3 group [OR = 1.065, 95%CI (1.028–1.104), *P *< 0.001]; the risk of MACE was increased by 65.9% in the SIRIQ2 and SIRIQ3 groups, respectively [OR = 1.659, 95%CI (1.068–2.578), *P *< 0.05] and 27.0% [OR = 1.270, 95%CI (1.152–1.401), *P *< 0.001]. In model 2, the risk of MACE was increased by 6.1% in the SIIQ3 group [OR = 1.061, 95%CI (1.018–1.105), *P *< 0.01]; the risk of MACE was increased by 115.3% in the SIRIQ2 and SIRIQ3 groups, respectively [OR = 2.153, 95%CI (1.251–3.705), *P *< 0.01] and 25.1% [OR = 1.251, 95%CI (1.123–1.394), *P *< 0.001] (Table 4).

**Table 4:** Regression analysis and trend test for each group under different adjusted models based on SII and SIRI triple quartiles.

Features	Crude	Model 1	Model 2
	OR (95%CI) *P*	OR (95%CI) *P*	OR (95%CI) *P*
SII/100 [median (range)]			
Q1[4.18 (<5.98)]	reference	reference	reference
Q2[8.23 (5.98–11.00)]	1.044 (0.912, 1.194)	1.023 (0.884, 1.184)	0.951 (0.805, 1.123)
Q3[16.38 (>11.00)]	1.079 (1.043, 1.115)***	1.065 (1.028, 1.104)***	1.061 (1.018, 1.105)**
*P* for trend	<0.001	<0.001	0.095
SIRI [median (range)]			
Q1[1.07 (<1.72)]	reference	reference	reference
Q2[2.52 (1.72–3.68)]	1.502 (1.018, 2.216)*	1.659 (1.068, 2.578)*	2.153 (1.251, 3.705)**
Q3[6.05 (>3.68)]	1.283 (1.173, 1.404)***	1.270 (1.152, 1.401)***	1.251 (1.123, 1.394)***
*P* for trend	<0.001	<0.001	<0.001

The effect values were magnified 100-fold using SII/100 due to insignificant effect values. Model 1 was adjusted for age, sex, diabetes mellitus, hypertensive disease, smoking history, and Killip class; model 2 was adjusted by adding LVEF, creatinine value, HDL-C, LDL-C, TG, TC, Glu, and Hcy variables to model 1. (Due to the different calculations of SII and SIRI, monocytes were included as covariates in the fully adjusted model in the regression analysis based on SII trimesters, and PLT was included as a covariate in the fully adjusted model in the regression analysis based on SIRI trimesters.).

SII, systemic inflammatory index; SIRI, System Inflammation Response Index; OR, hazard ratio; 95% CI, 95% confidence interval.

* *P *< 0.05.

** *P *< 0.01.

*** *P *< 0.001.

*P *< 0.05 was considered statistically significant.

## 4. Discussion

In the present study, we found that high levels of SII and SIRI were strongly associated with the occurrence of postinfarction in-hospital MACE in patients with AMI, and this association persisted after adjusting for confounding factors such as age, sex, history, and relevant clinical indicators. In the subsequent prediction model, we found that SII and SIRI had high predictive values for MACE, and their sensitivity and specificity for predicting MACE were 56.8%, 71.6% and 74.2%, 62.0% when SII > 1,085.55, SIRI > 2.96, respectively. In further correlation analysis, it was demonstrated that SII and SIRI were linearly correlated with GRACE; we also found that SIRI and Gensini, QTc, and GRACE were linearly correlated in patients who developed in-hospital MACE. The high levels of SII and SIRI predict the stimulation of immune inflammation in AMI patients, which also suggests that we should pay great attention to improving the internal immune environment of the body in AMI patients after surgery.

AMI is an end-stage manifestation in patients with coronary heart disease, intrinsically caused by coronary atherosclerosis. Atherosclerosis is an inflammatory disease of the arteries, and the immune-inflammatory system plays a critical role in the various stages of vessel wall damage, lipid deposition, fibrous cap formation, atheromatous plaque rupture and thrombosis in atherosclerotic cardiovascular disease (19–21). Previous studies have elucidated the inflammatory factors and inflammatory signaling pathways that contribute to atherosclerosis (22, 23). It has been shown that elevated C-reactive protein, interleukin-1, and tumour necrosis factor are associated with pro-inflammatory and atherogenic atherosclerosis (24), and mononuclear phagocytes can advance the progression of all stages of atherosclerosis (22). Therefore, further study of inflammatory agents is crucial in patients who develop severe MACE. SII and SIRI integrate three independent leukocyte subpopulations and platelets, reflecting the interaction of immunity and inflammation (25). The elevation of SII and SIRI indicates an increase in neutrophils, platelets, and monocytes and a decrease in lymphocytes, indicating a combination of non-specific inflammatory and adaptive immune response impairment of the organism at the cellular layer (26). A large cohort study recently revealed that SII and SIRI, which represent chronic low-grade inflammation, are significantly associated with cardiovascular and all-cause mortality across the U.S. population (25). A cohort study from Kailuan included 85,154 subjects and showed that higher SII and SIRI were associated with an increased risk of hemorrhagic stroke and ischemic stroke at a 10-year follow-up interval (27). An analysis of the association of SII and SIRI with postoperative mortality after off-pump coronary artery bypass surgery from Polish scholars, who included 538 patients (median follow-up 4.7 ± 1.7 years), revealed that SIRI was associated with risk of disease progression after COX regression modelling and adjustment for confounders, whereas SII was not independently correlated following model adjustment (28). A retrospective cohort study in China on SII, SIRI and long-term outcomes in type B aortic coarctation identified a significant association between SIRI and prognostic adverse aortic events in patients when the investigators adjusted the COX regression model. At the same time, SII was no longer statistically significant under the fully revised model (29). Nevertheless, several cross-sectional studies have found significant associations between SII and hypertension (30), hyperlipidemia (31), and abdominal aortic calcification (32). This study further revealed the predictive value of SII and SIRI for AMI patients by investigating the correlation between SII and SIRI and post-myocardial infarction MACE in AMI patients while including the combined risk factors of Gensini, QTc, and GRACE.

The success of coronary reperfusion therapy in AMI patients usually means a high survival rate. And the prognosis management of AMI patients after reperfusion treatment is equally highly valued. In the present study, we revisited the importance of immunoinflammatory factors in AMI. Previous studies have indicated that targeted anti-inflammatory therapy can significantly reduce the recurrence of cardiovascular events (33). Therefore, individualized anti-inflammatory therapy and immunotherapy for AMI are potentially beneficial.

## 5. Conclusion

The present study revealed the correlation between the Systemic Immunoinflammatory Index (SII) and the Systemic Inflammatory Response Index (SIRI), and in-hospital cardiovascular adverse events in patients with acute myocardial infarction. Elevated levels of SII and SIRI increase the likelihood of in-hospital adverse outcomes in patients with acute myocardial infarction. In addition, this study analyzed the correlation between SII, SIRI and the potential clinical risk of post-myocardial infarction Gensini, QTc, and GRACE in patients with myocardial infarction. The present investigation demonstrates the value of early prevention of inflammation in predicting patients with acute myocardial infarction and the need for a large cohort study to improve the prognosis of patients with acute myocardial infarction.

## Data Availability

The raw data supporting the conclusions of this article will be made available by the authors, without undue reservation.
