# Supplementary material for: Association of systemic immune inflammation index and system inflammation response index with clinical risk of acute myocardial infarction
Source: Front Cardiovasc Med. 2023 Aug 30;10:1248655. doi: 10.3389/fcvm.2023.1248655 (PMC10498290; doi:10.3389/fcvm.2023.1248655)
Supplement: Supplementary file 1 [file Table1.docx]

**Table S1 Comparison between the two groups based on the propensity matching method**

| **Features** | **MACE（n=61）** | **NMACE(n=61)** | **X^2^/Z/T value** | ***P*** |
| --- | --- | --- | --- | --- |
| Age (years) | 66.44±13.41 | 66.52±14.72 | 0.032 | 0.974 |
| Killip（II-IV） | 24（39.34） | 26（42.62） | 0.136 | 0.713 |
| LVEF | 56.00(50.50, 60.50) | 57.00(48.00, 61.00) | -0.182 | 0.856 |
| Creatinine (μmol/L) | 78.08±22.37 | 74.95±24.02 | -0.744 | 0.458 |
| HGB(g/L) | 128.51±17.56 | 129.74±22.44 | 0.338 | 0.736 |
| QTc (ms) | 450.33±33.06 | 449.90±25.33 | -0.080 | 0.936 |
| Glu | 6.81(5.57, 8.83) | 6.45(5.49, 8.63) | -0.556 | 0.578 |
| Hcy | 17.20(12.54, 20.70) | 13.60(10.51,18.3) | -1.600 | 0.110 |
| SII | 1069.72(603.07, 1907.75) | 1476.00(993.40, 1828.29) | -2.256 | 0.024 |
| SIRI | 3.56(2.37, 6.38) | 4.06(2.60, 6.19) | -0.509 | 0.610 |

Killip class: clinical classification of heart failure; LVEF: left ventricular ejection fraction; HGB: haemoglobin; Glu: fasting glucose; Hcy: homocysteine; SII: Systemic Immune Inflammation Index; SIRI: System Inflammation Response Index.
